# Age-dependent bone mineral density responses to gender-affirming hormone therapy in transgender individuals: a one-year prospective study

**DOI:** 10.1007/s40618-025-02675-5

**Published:** 2025-08-04

**Authors:** Chiara Ceolin, Martina Dall’Agnol, Giulia Termini, Mario Virgilio Papa, Giulia Casali, Anna Bertocco, Alberto Scala, Sandro Giannini, Alberto Ferlin, Giuseppe Sergi, Andrea Garolla, Marina De Rui

**Affiliations:** 1 Geriatrics Division, Department of Medicine (DIMED), University of Padua, Padua, Italy; 2 Department of Neurobiology, Care Sciences and Society, Aging Research Center, Karolinska Institutet and Stockholm University, Stockholm, Sweden; 3 Unit of Andrology and Reproductive Medicine, Department of Medicine (DIMED), University of Padua, Padua, Italy; 4 Department of Medicine, Clinica Medica 1, University of Padua, Padua, Italy

**Keywords:** Transgender, Gender-Affirming hormone therapy, Gender incongruence, Bone, BMD

## Abstract

**Purpose:**

Evidence on the skeletal effects of gender-affirming hormone therapy (GAHT) in transgender individuals remains limited, especially across age groups. Individuals assigned male at birth (AMAB) often show reduced bone mineral density (BMD) even before GAHT, whereas findings in those assigned female at birth (AFAB) are more variable. Given the key role of adolescence and early adulthood in peak bone mass, timely skeletal assessment is essential. This study compared BMD before and after one year (1-y) of GAHT to age-matched cisgender controls.

**Methods:**

Prospective observational study involving 269 adults (162 transgender and 107 cisgender controls) conducted at the University Hospital of Padua (January 2020-November 2024). Dual-energy X-ray absorptiometry (DXA) was performed at baseline and after 1-y of GAHT.

**Results:**

After 1-y of GAHT, in AMAB individuals, lumbar spine BMD significantly increased (from 0.97 ± 0.16 to 1.02 ± 0.14 g/cm², *p* < 0.001), particularly in those under 20 years. AFAB individuals experienced a modest but significant reduction in femoral neck BMD (from 0.81 ± 0.12 to 0.79 ± 0.13, *p* < 0.05), especially in the 20–30-year age group. Age-stratified analyses revealed that younger participants showed greater BMD improvements, while those over 20 exhibited stable or declining values. Linear regression confirmed age as an independent predictor of BMD change, with older age associated with reduced skeletal responsiveness to GAHT at key femoral sites.

**Conclusions:**

GAHT has variable effects on bone health, influenced by age and sex assigned at birth. Early initiation may favor bone accrual, especially in AMAB individuals, while AFAB individuals may require closer monitoring for site-specific bone loss during testosterone therapy.

**Supplementary Information:**

The online version contains supplementary material available at 10.1007/s40618-025-02675-5.

## Introduction

Gender identity refers to an individual’s internal sense of being a woman, a man, or another gender (non-binary). Individuals whose gender identity corresponds to their assigned sex at birth are defined as cisgender, while those whose gender differs from the one assigned at birth are referred to as transgender (TGD) [1, 2]. Recognizing the broad spectrum of gender identities, in research contexts, the acronyms AFAB (assigned female at birth) and AMAB (assigned male at birth) are used to categorize individuals based on sex assignment. Gender-affirming hormone therapy (GAHT) is a cornerstone of gender-affirming care and involves estrogen-based regimens for AMAB individuals and testosterone therapy for AFAB individuals [3]. Although its systemic effects are being increasingly investigated, current evidence on skeletal outcomes remains limited and inconsistent [4, 5]. Given that bone is both sexually dimorphic and hormonally regulated, GAHT may significantly influence bone metabolism [6, 7]. In young people, estrogens primarily preserve bone mass by inhibiting osteoclastic bone resorption, while androgens contribute to periosteal apposition [8, 9]. The onset of menopause in cisgender women or age-related androgen decline in men leads to accelerated bone loss and heightened risk of osteoporosis [10].

In TGD populations, prior studies have demonstrated divergent effects of GAHT on bone health. AMAB individuals generally present with lower bone mineral density (BMD) even prior to initiating GAHT, compared to cisgender men [11, 12]. Some data suggest that GAHT may stabilize or improve BMD in AMAB individuals, particularly at the lumbar spine, whereas results in AFAB individuals are more variable and site-dependent [13, 14]. However, to our knowledge, only one study has investigated how BMD evolves across different age groups in TGD individuals, both before and after the initiation of GAHT [15]. This knowledge gap is particularly concerning given the critical role of adolescence and early adulthood in achieving peak bone mass [16]. The age at which GAHT is initiated may significantly influence skeletal development during gender affirming treatments. A deeper understanding of these dynamics is essential not only to clarify the physiological effects of hormone therapy, but also to guide clinical monitoring and optimize long-term bone health in TGD individuals receiving GAHT.

This study aims to provide a comprehensive evaluation of BMD in TGD individuals before and after one year of GAHT. Uniquely, it incorporates an age-stratified design, allowing us to explore whether skeletal trajectories during gender affirming treatments reflect delayed peak bone mass acquisition. To our knowledge, this is one of the first studies to systematically assess age-dependent differences in skeletal response to hormone therapy.

## Materials and methods

### Study population

This prospective observational study included 269 adult participants: 162 TGD individuals, and 107 cisgender controls. TGD individuals were recruited from the Regional Reference Center for Gender Incongruence at the Andrology and Reproductive Medicine Unit of the University Hospital of Padua (Italy) between January 2020 and November 2024. Before the initiation of hormone therapy, all TGD participants underwent a baseline osteometabolic assessment performed by a team of specialists from the Geriatrics Unit of the same hospital. The inclusion criteria for the TGD group have been previously described [16, 17]. In brief, participants were eligible if they had a diagnosis of gender dysphoria, no current or past exposure to GAHT, no history of gender-affirming surgery, were between 16 and 50 years of age, and had a body mass index (BMI) between 19 and 35 kg/m². Exclusion criteria included: previous or current use of GAHT; history of gender-affirming surgery; BMI < 19 or > 35 kg/m²; use of medications known to affect bone metabolism (e.g., chronic corticosteroids, antiepileptics, bisphosphonates); and presence of medical conditions associated with secondary osteoporosis or impaired bone health (e.g., malabsorptive disorders, endocrine bone diseases such as hyperparathyroidism or osteogenesis imperfecta).

Cisgender control participants were randomly selected from a population of university students, with stratification by age and sex to ensure demographic comparability with the TGD group.

The study protocol was approved by the Ethics Committee for Clinical Research of the Province of Padua (approval no. 0025087) and conducted in accordance with the principles of the Declaration of Helsinki. Written informed consent was obtained from all participants prior to enrollment.

### Data collection

Clinical, laboratory, and densitometric assessments were performed as previously described [17, 18]. Briefly, data were obtained from medical records and DXA scans were conducted using a Hologic QDR 4500 W system. Moreover, data about Fracture Risk Assessment (FRAX) was collected. The FRAX tool is a computerized algorithm used to estimate the 10-year probability of hip fracture and/or major osteoporotic fracture (e.g., vertebral, humeral, forearm), based on femoral neck BMD and individual risk factors in persons aged 40–90 years. These factors include sex, weight (kg), height (cm), previous fractures, family history of fracture, smoking, alcohol intake, glucocorticoid use, rheumatoid arthritis, and secondary osteoporosis (e.g., type 1 diabetes, osteogenesis imperfecta, hyperthyroidism, hypogonadism, premature menopause, liver disease) [19].

### Endpoints

The primary endpoint of the study was the change in BMD at the lumbar spine, femoral neck, and total hip after one year of GAHT. Secondary endpoints included the association between age and BMD variation, as well as comparison of BMD trajectories between transgender individuals and age-matched cisgender controls.

### Statistical analysis

Categorical variables were expressed as frequencies and percentages; continuous quantitative variables were expressed as mean ± standard deviation or median (interquartile range). The Shapiro-Wilk test was used to assess normality of continuous variables. To compare variables between cisgender and transgender individuals, or between pre- and post-GAHT values, the Mann-Whitney and Kruskal-Wallis tests were used for continuous variables. Multiple linear regression models with a forward stepwise procedure were used to examine independent associations between BMD values after one year of GAHT and potential covariates previously identified as significant (*p* < 0.20) in univariate analyses. Multicollinearity was assessed using the Variance Inflation Factor (VIF), with a cut-off of 2 for exclusion. Multivariate models were adjusted for age, body mass index (BMI), smoking status, and circulating vitamin D levels. Statistical significance was set at *p* ≤ 0.05. Analyses were performed using IBM SPSS Statistics version 29 (IBM Corp., Armonk, NY) and R version 4.1.1 (2021-08-10) (R Foundation for Statistical Computing, Vienna, Austria).

## Results

### Baseline data

Table 1 summarizes the demographic and hormonal characteristics of the study population. The TGD group included 96 AFAB (59.2%) and 66 AMAB (40.7%) individuals. These were compared with a cisgender peer control group: 56 cisgender AFAB and 51 cisgender AMAB young adults. No significant differences were observed in hormone profiles between TGD and cisgender groups at baseline.

**Table 1:** Demographic, and hormonal characteristics of transgender and cisgender participants at baseline

Variables	AFAB trans (*n* = 96)	AFAB cis (*n* = 56)	*p*-value	AMAB trans (*n* = 66)	AMAB cis (*n* = 51)	*p*-value
Age [years]	24.2 **±** 5.9	25.7 **±** 3.8	0.10	24.8 **±** 7.6	25.8 **±** 4.2	0.21
BMI [kg/m^2^]	*24.51* ***±*** *7.22*	*21.79* ***±*** *2.64*	*0.01*	*21.80* ***±*** *3.60*	*23.33* ***±*** *2.48*	*0.01*
Active smokers [%]	32 (33.3%)	6 **(**10.7%)	0.07	17 **(**25.7%)	7 (13.7%)	0.80
**Hormonal profile**						
LH [U/L]	6.79 (4.43;9.75)	9.62 (5.87;13.27)	0.10	4.64 (3.3;5.6)	5.97 (4.71;6.67)	0.10
FSH [U/L]	5.61 (3.47;6.27)	5.51 (4.28;6.26)	0.33	4.26 (1.76;5.58)	2.42 (2.09;3.9)	0.43
Estradiol [pmol/L]	232.48 (158.19;539.28)	247.89 (182.9;422.3)	0.27	110.89 (84.37;131.29)	93.63 (74.07;135.59)	0.75
Testosterone [nmol/L]	1.17 (0.79;1.7)	1.51 (1.12;1.68)	0.15	17.78 (14.03;24.67)	19.81 (14.77;20.32)	0.81

*Notes*: Values are expressed as mean ± standard deviation (SD) or median (IQR) or count (%)

*Abbreviations*: AFAB = Assigned Females At Birth; AMAB = Assigned Males At Birth; Trans = Transgender; Cis = Cisgender; BMI = Body Mass Index; LH, Luteinizing Hormone; FSH, Follicle-Stimulating Hormone

**Fig. 1:**
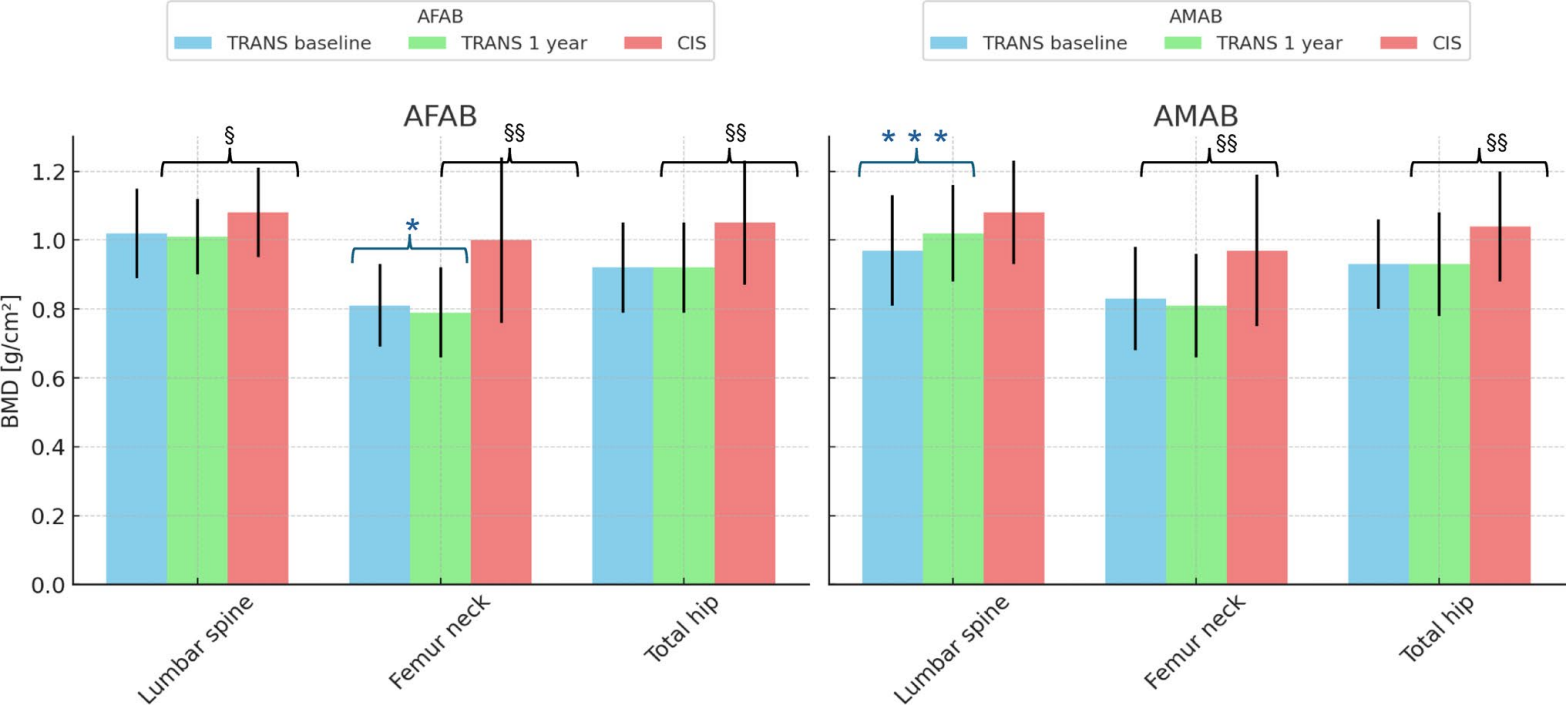
Changes in Bone Mineral Density Values After One Year of GAHT in transgender people. *Abbreviations*: TRANS: Transgender; CIS: Cisgender; AFAB: Assigned Females At Birth; AMAB: Assigned Males At Birth; BMD: Bone Mineral Density. Statistical significance: *Indicates comparison between transgender individuals at baseline and after one year of GAHT: *p < 0.05, ***p < 0.001. ^§^Indicates comparison between transgender individuals after one year of GAHT and cisgender controls: ^§^*p* < 0.05, ^§§^ *p* < 0.01, ^§§§^*p* < 0.001. *Notes*: In AFAB participants, no significant changes were observed at the lumbar spine and total hip, while a slight but significant reduction emerged at the femoral neck. When compared to cisgender AFAB controls, BMD values remained significantly lower across all sites. In contrast, AMAB individuals showed a significant increase in lumbar spine BMD after one year of estrogen therapy, with values approaching those of cisgender AMAB controls. However, BMD at the femoral neck and total hip did not significantly change and remained below cisgender reference values

Bone metabolism-related variables after one year of GAHT in TGD people, compared to the cisgender population, are reported in Table 2. At baseline, TGD individuals exhibited significantly lower BMD across all skeletal sites compared to cisgender controls. Specifically, TGD AFAB participants had reduced BMD values at the lumbar spine (1.01 ± 0.11 vs. 1.08 ± 0.13 g/cm²), femoral neck (0.81 ± 0.11 vs. 1.00 ± 0.24), and total femur (0.93 ± 0.12 vs. 1.04 ± 0.18; all *p* < 0.001) compared to cisgender peers. In TGD AMAB participants, BMD was significantly lower especially at the femur sites. Regarding calcium-phosphorus metabolism, vitamin D levels were lower in TGD individuals, with statistical significance reached particularly in the AMAB group. FRAX scores showed a trend toward higher estimated 10-year fracture risk in the TGD groups, for both hip and major osteoporotic fractures, although these differences were not statistically significant (Supplementary Fig. 1). During the first year of GAHT, TGD AMAB participants were treated primarily with cyproterone acetate as an antiandrogen, with spironolactone used less frequently. Estrogens were administered via transdermal sprays, gels, or patches, while oral estradiol valerate was rarely used. TGD AFAB individuals received injectable testosterone (undecanoate or enanthate) or transdermal testosterone preparations. Furthermore, vitamin D supplementation was administered appropriately, based on the observed deficiency and in line with current guidelines [20]. Specifically, vitamin D supplementation was prescribed when serum levels fell below 50 nmol/L, in line with national recommendations. Cholecalciferol was administered either as a 25,000 IU weekly oral dose or as a 100,000 IU monthly dose, depending on severity and clinical judgment. Supplementation was continued for a minimum of 3 months and re-evaluated based on follow-up levels. After one year of GAHT, skeletal responses differed by sex assigned at birth. Among AFAB individuals, BMD remained stable at the lumbar spine and total femur, while a modest but significant reduction occurred at the femoral neck (from 0.81 ± 0.12 to 0.79 ± 0.13, *p* < 0.05). Notably, BMD values at both femoral sites remained significantly lower compared to age-matched cisgender peers, indicating persistent deficits despite hormone treatment. In AMAB participants, a significant increase in lumbar spine BMD was observed (from 0.97 ± 0.16 to 1.02 ± 0.14 g/cm², *p* < 0.001). However, BMD values remained below those of young cisgender men (Fig. 1). In terms of biochemical findings, both TGD cohorts showed increased vitamin D levels during the first year of GAHT, reaching values comparable to those of their cisgender counterparts, as previously documented in our earlier study [18].

**Table 2:** Bone metabolism-related variables after one year of GAHT in transgender people, compared to the cisgender population

Variables	AFAB trans (*n* = 96)	AFAB trans 1-y GAHT	AFAB cis (*n* = 56)	AMAB trans (*n* = 66)	AMAB trans 1-y GAHT	AMAB cis (*n* = 51)
*Densitometric values*						
BMD lumbar spine [g/cm2]	1.02 ± 0.13	1.01 ± 0.11	1.08 ± 0.13^§^	0.97 ± 0.16	1.02 ± 0.14 ***	1.08 ± 0.15
BMD femur neck [g/cm2]	0.81 ± 0.12	0.79 ± 0.13*	1.00 ± 0.24^§§§^	0.83 ± 0.15	0.81 ± 0.15	0.97 ± 0.22^§§^
BMD total hip [g/cm2]	0.92 ± 0.13	0.92 ± 0.13	1.05 ± 0.18^§§^	0.93 ± 0.13	0.93 ± 0.15	1.04 ± 0.16^§§^
z-score lumbar spine	−0.19 ± 1.07	−0.20 ± 0.98	−0.18 ± 0.90	−0.99 ± 1.13	−0.72 ± 1.30	−0.60 ± 1.12
z-score femur neck	−0.35 ± 1.03	−0.50 ± 1.17**	−0.16 ± 0.88^§^	−0.94 ± 1.32	−0.60 ± 0.97 ***	−0.52 ± 0.99
z-score total hip	−0.12 ± 1.00	−0.12 ± 1.11	−0.07 ± 0.81	−0.72 ± 0.88	−0.59 ± 0.91 ***	−0.29 ± 0.89^§§^
*Calcium-phosphor metabolism*						
Calcium [mmol/L]	2.53 **±** 0.14	2.45 **±** 0.20	2.40 **±** 0.06	2.41 **±** 0.09	2.41 **±** 0.06	2.39 **±** 0.05
Phosphorus [mmol/L]	1.01 **±** 0.41	1.35 **±** 0.04	2.39 **±** 0.06***	1.00 **±** 0.19	0.99 **±** 0.18	1.02 **±** 0.16
PTH [ng/L]	31.80 (24.10–36.80)	24.95 (18.87–36.47)	24.50 (16.80–35.20)	25.30 (19.10–35.80)	32.20 (24.65–37.35)	28.10 (25.20–36.80)
Vitamin D [nmol/L]	44.00 (34.50–68.10)	65.90 (44.25–118.00)*	54.20 (37.10–74.80)	41.30 (28.10–68.50)	66.80 (52.47–115.90)*	57.40 (41.20–81.90)**

*Notes*: Values are expressed as mean ± standard deviation (SD) or median (IQR)

*Abbreviations*: AFAB = Assigned Females At Birth; AMAB = Assigned Males At Birth; Trans = Transgender; Cis = Cisgender; BMD = Bone Mineral Density; PTH, Parathyroid Hormone

Statistical significance: * indicates comparison between transgender individuals at baseline and after one year of GAHT: *p < 0.05, ***p < 0.001

^§^Indicates comparison between transgender individuals after one year of GAHT and cisgender controls: ^§^*p* < 0.05, ^§§^ *p* < 0.01, ^§§§^*p* < 0.001

### Age-Stratified analysis

**Fig. 2:**
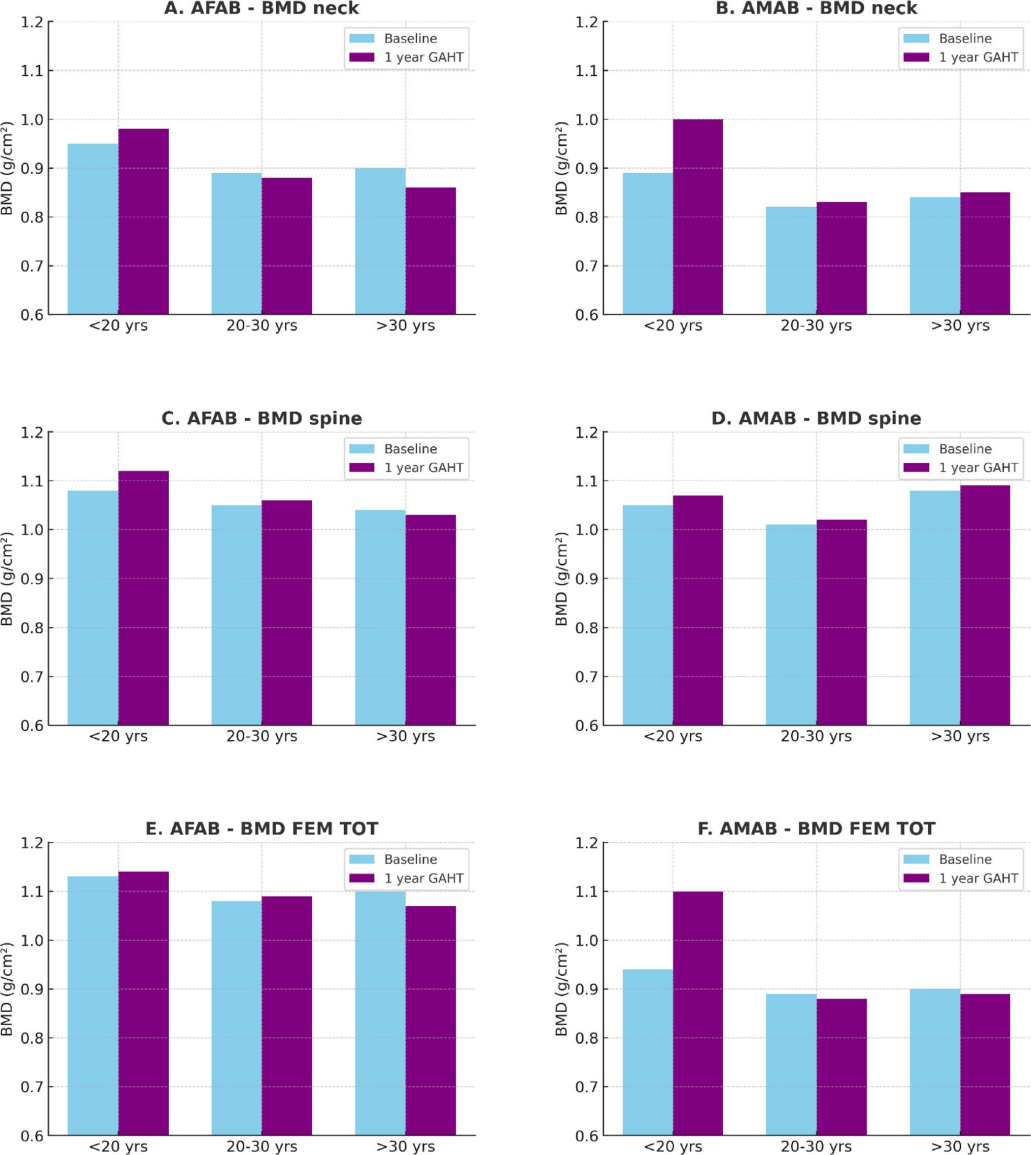
Changes in Bone Mineral Density (BMD) at the Lumbar Spine, Femoral Neck, and Total Femur in Transgender Individuals by Age Group, at Baseline and After One Year of GAHT

Age significantly influenced skeletal outcomes. Among AFAB individuals, 21 were under 20 years old, 55 were between 20 and 30 years, and 20 were over 30. In AMAB individuals, 20 were under 20, 31 between 20 and 30, and 15 over 30. In AFAB individuals under 20 years old, BMD at the total femur improved significantly after one year of GAHT (*p* = 0.02), whereas in older age groups no meaningful change was observed. In AMAB individuals under 20, baseline BMD was reduced across all sites, but showed consistent improvement after one year of GAHT (*p* = 0.01), particularly at the spine. In contrast, individuals aged 20–30 and > 30 exhibited stable BMD levels post-GAHT, with no statistically significant changes (see Fig. 2).

### Age as a predictor of BMD changes after GAHT

Linear regression analyses confirmed age as a key determinant of skeletal response. In AFAB individuals, each additional year of age was associated with a significant reduction in femoral neck BMD (β = −0.46, *p* = 0.02) and total hip BMD (β = −0.60, *p* = 0.005) variation. Among AMAB participants, older age was similarly associated with reduced BMD variation at the femoral neck (β = −0.41, *p* = 0.02) (Table 3).

**Table 3:** Linear regression analysis of predictors of BMD changes after one year of GAHT

	AFAB		AMAB	
	Univariate Beta (95% IC, *p*-value)	Multivariate Beta (95% IC, *p*-value)	Univariate Beta (95% IC, *p*-value)	Multivariate Beta (95% IC, *p*-value)
BMD lumbar spine [g/cm^2^]				
Age [years]	−0.18 (−0.01; 0.005, p = 0.33)	–	0.26 (−0.003; 0.01, p = 0.22)	–
R^2^	0.23		0.33	
BMD femur neck [g/cm^2^]				
Age [years]	−0.38 (−0.02; −0.001, p = 0.03)	**−0.46 (−0.02; −0.003**, **p = 0.02)**	−0.40 (−0.02; 0.001, p = 0.05)	**−0.41 (−0.009; −0.01**, **p = 0.02)**
R^2^	0.67		0.68	
BMD total hip [g/cm^2^]				
Age [years]	−0.25 (−0.01; 0.003, p = 0.18)	**−0.60 (−0.03; −0.006**, **p = 0.005)**	−0.28 (−0.01; 0.003, p = 0.19)	−0.22 (−0.08; 0.02, p = 0.24)
R^2^	0.40		0.52	

*Abbreviations*: AFAB = Assigned Females At Birth; AMAB = Assigned Males At Birth; BMD = Body Mineral Density; FMI = Fat Mass Index; ASMMI = Appendicular Skeletal Muscle Mass Index. Models were adjusted for age, body mass index (BMI), smoking habits, and serum vitamin D levels. Significant findings are in bold

## Discussion

This study provides a comprehensive analysis of bone health in TGD individuals before and after one year of GAHT, highlighting age- and sex-specific differences in skeletal response. At baseline, BMD was markedly lower in TGD individuals compared to age-matched cisgender controls, with AMAB participants showing the most pronounced deficits. After one year of GAHT, AMAB individuals experienced a significant increase in lumbar spine BMD, particularly those under 20 years of age, suggesting a favorable response during adolescence. In contrast, AFAB individuals showed a modest decline in femoral neck BMD, especially in the 20–30 age group, indicating a potential vulnerability to testosterone-related bone loss. These findings underscore the importance of the timing of GAHT initiation in relation to peak bone mass accrual, suggesting that earlier hormonal intervention—particularly before the completion of skeletal maturation—may offer a more favorable bone health trajectory, while later initiation might require closer monitoring and preventive strategies.

The age-stratified design of this study adds a novel dimension to the understanding of how skeletal tissue responds to hormonal modulation across different stages of young adulthood. Notably, these results share both similarities and divergences with the findings reported by Wiepjes et al. [15]. In their large cohort study, TGD AMAB showed significant increases in BMD at the lumbar spine, femoral neck, and total hip after one year of GAHT. Similarly, our data confirm a significant gain in lumbar BMD in AMAB individuals, with a more pronounced response observed in those aged 18–20 years, consistent with their findings. This finding supports the concept of a “window of skeletal plasticity” during adolescence, a phase in which bone tissue demonstrates heightened responsiveness to hormonal stimuli [21]. Estrogen appears to exert a particularly anabolic effect during this period, likely through endocortical apposition and increased trabecular density [22]. In contrast, AMAB individuals over 20 showed only a stabilization of BMD without significant gains, suggesting that once skeletal maturation is complete, hormonal interventions may have a more limited capacity to induce structural changes. However, some differences emerged in the response among AFAB participants. While Wiepjes et al. reported increases in lumbar and total hip BMD and stable values at the femoral neck in TGD AFAB, our study observed a slight but statistically significant decline in femoral neck BMD, particularly in the 20–30 age range. This discrepancy may reflect differences in population characteristics, GAHT regimens, or timing of treatment initiation relative to skeletal maturity. Interestingly, in the Wiepjes study, AFAB individuals over 50 years—many of whom were postmenopausal—demonstrated greater gains in lumbar BMD, likely due to the reintroduction of protective androgenic effects. Additionally, trans men aged 18–20 showed greater increases in total hip BMD compared to those aged 20–49, supporting the hypothesis that earlier initiation of GAHT may yield more favorable skeletal outcomes. The mechanism underlying this pattern is likely multifactorial. Testosterone, while capable of stimulating periosteal bone formation [23], may lack the protective antiresorptive effects of estrogen, particularly in cortical-rich skeletal regions such as the femoral neck and total hip. This mismatch could lead to an imbalance in bone remodeling, potentially compromising microarchitectural integrity over time—especially in the absence of preventive strategies or adequate monitoring. In addition, experimental data suggest that during the early stages of periosteal expansion, testosterone may induce increased cortical porosity before compensatory thickening occurs, a process well-documented in adolescent males [24]. While this effect contributes to larger bone cross-sectional area and strength in cisgender men over time, its short-term implications in transmasculine individuals initiating GAHT after skeletal maturation remain unclear. Furthermore, the bone tissue of individuals exposed to testosterone may exhibit reduced responsiveness to mechanical loading compared to estrogen-dominant physiology, as suggested by lower sclerostin suppression during exercise and a lower muscle-to-BMD efficiency ratio observed in cisgender males [25]. Given that trans men experience gains in lean mass during the first year of GAHT, yet generally do not reach cis male levels [18], the mechanical stimulus exerted on the skeleton may be insufficient to fully counterbalance androgen-induced remodeling changes. This complex interaction could explain the subtle but measurable decline in femoral neck BMD observed in our younger adult AFAB cohort.

These findings raise important clinical considerations. First, they suggest that TGD individuals may be at increased risk of suboptimal bone health even before the initiation of GAHT. In AMAB individuals, low baseline BMD likely reflects a combination of biological, behavioral, and social factors that impair bone accrual during adolescence. These may include delayed or suppressed puberty, inadequate nutritional intake, low physical activity levels, and psychosocial stress—all of which are prevalent among TGD youth and known to adversely affect bone development [17, 26]. Rather than reflecting premature bone loss, these deficits may represent a missed opportunity for optimal peak bone mass acquisition, a concept supported by previous studies documenting smaller bone size and reduced areal BMD in AMAB individuals compared to cisgender controls [27]. Second, the observation that BMD remained below normative values in AMAB individuals even after one year of GAHT suggests that estrogen therapy may support stabilization and partial recovery, but may not be sufficient to fully restore skeletal mass once the accrual window has closed. In this context, the timing of GAHT initiation emerges as a modifiable factor, with earlier access—particularly during adolescence—potentially offering more pronounced skeletal benefits. For AFAB individuals, the picture is more complex. The modest decline in femoral neck BMD observed in young adults may reflect a transitional phase of skeletal remodeling rather than true bone loss, potentially driven by androgen-induced changes in cortical architecture and suboptimal mechanical loading. These findings emphasize the need for ongoing BMD monitoring and targeted preventive strategies, particularly in those initiating GAHT after peak bone mass has been reached.

These findings are consistent with prior research reporting lower BMD values in TGD AMAB individuals at both the lumbar spine and hip [16, 26–30]. A prospective observational study noted reduced areal BMD and smaller bone dimensions compared to cisgender controls [31], and similar trends have been reported in Norwegian cohorts [32]. Notably, FRAX scores did not differ significantly between groups, despite measurable differences in BMD. Although our study population was younger than the cohort on which FRAX was originally validated, this discrepancy may indicate that standard fracture risk assessment tools are not well calibrated for use in TGD individuals. FRAX algorithms are based on cisgender reference data and do not account for hormonal regimens, duration of therapy, or sex assigned at birth. As a result, their predictive validity in this context is uncertain, and dedicated tools may be required to assess fracture risk in transgender individuals more accurately.

Taken together, these findings suggest that skeletal health in TGD individuals is shaped by both hormonal therapy and the timing of its initiation, alongside developmental and lifestyle factors. From a clinical perspective, these results support early, individualized bone health assessment, including baseline BMD screening prior to GAHT—particularly in AMAB individuals over age 20 and AFAB individuals with additional risk factors. Lifestyle measures such as weight-bearing exercise, adequate calcium and vitamin D intake, and smoking cessation remain essential. Periodic BMD monitoring should be considered during follow-up to detect early changes and guide interventions if needed. Finally, bone health should be integrated into holistic models of TGD care. To this end, multicenter longitudinal studies with larger samples, extended follow-up, and comprehensive data collection are needed to clarify bone adaptation patterns, define reference standards, and inform evidence-based guidelines for skeletal preservation across the gender transition process.

### Limitations and strengths

This study has several limitations. First, the one-year follow-up period is relatively short to fully evaluate the long-term impact of GAHT on skeletal health. In particular, outcomes such as fracture risk and the attainment of peak bone mass require extended longitudinal observation beyond the timeframe of this study. Second, due to its observational design, the reported associations should be interpreted as correlational rather than causal. Third, small subgroup sizes—particularly among older participants—may increase the risk of overfitting in regression models, and the high R² values should therefore be interpreted with caution. Larger and more powered cohorts are needed to confirm these findings. Furthermore, although potential confounders such as dietary intake, menstrual history, physical activity, eating disorders, and adherence to therapy may affect BMD trajectories, these variables were not consistently available or sufficiently represented to allow meaningful stratification. These limitations should be addressed in future studies to enhance model robustness and generalizability. While objective hormone levels were regularly monitored, no formal tools (e.g., validated adherence scales) were used to assess compliance. Nevertheless, close clinical follow-up and biochemical monitoring likely ensured adequate therapeutic exposure. Additionally, the use of a control group composed of university students may introduce selection bias due to socioeconomic or behavioral differences compared to the transgender cohort. This could influence lifestyle-related factors relevant to bone health and should be considered when interpreting group comparisons. Finally, the FRAX algorithm has not been validated for individuals under 40 years of age or for transgender populations, limiting its predictive value in this context.

Despite these limitations, the study presents notable strengths. Its longitudinal design allows direct evaluation of GAHT effects over time, and the age-stratified approach offers novel insight into developmental differences in skeletal responsiveness.

## Conclusion

In summary, our study demonstrates that TGD AMAB individuals exhibit significantly reduced BMD prior to initiating GAHT compared to both age-matched cisgender peers. While GAHT results in a modest improvement in BMD after one year, this increase does not achieve normative values for healthy young adults. In contrast, AFAB individuals show an age-related decline in BMD following GAHT, likely due to testosterone’s limited capacity to counterbalance estrogen deficiency, raising concerns about the risk of early-onset bone loss. Further research involving larger samples and extended follow-up is necessary to confirm these findings, elucidate underlying biological mechanisms, and optimize bone health strategies in TGD populations.

## Supplementary Information

Below is the link to the electronic supplementary material.

Supplementary Material 1

## Data Availability

The datasets generated during and/or analysed during the current study are not publicly available due to privacy reasons but are available from the corresponding author on reasonable request.
