# Supplementary material for: Age-dependent bone mineral density responses to gender-affirming hormone therapy in transgender individuals: a one-year prospective study
Source: J Endocrinol Invest. 2025 Aug 4;48(12):2879–88. doi: 10.1007/s40618-025-02675-5 (PMC12640344; doi:10.1007/s40618-025-02675-5)
Supplement: Supplementary file 1 — Supplementary Material 1 [file 40618_2025_2675_MOESM1_ESM.docx]

**Supplementary Figure 1. Ten-Year Estimated Fracture Risk (FRAX Scores) in Transgender and Cisgender Individuals.**


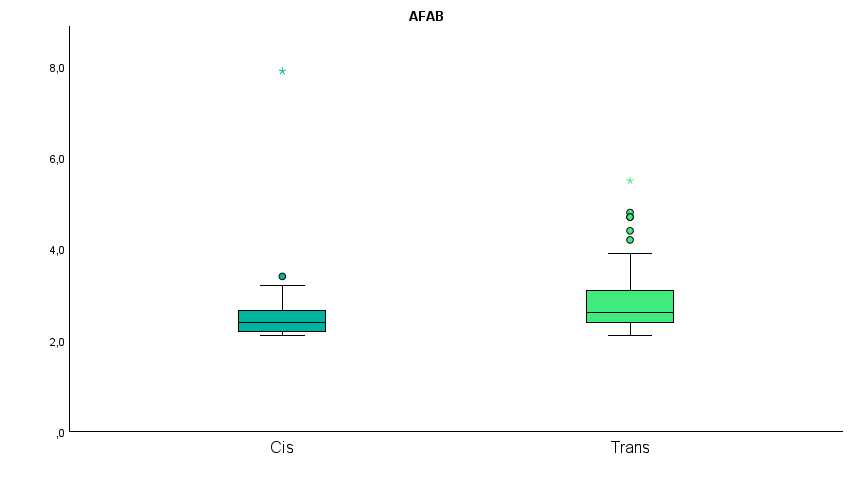

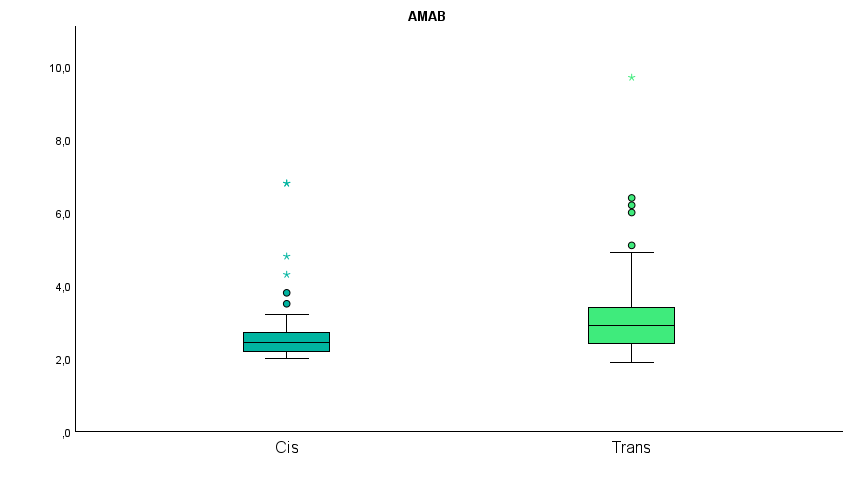

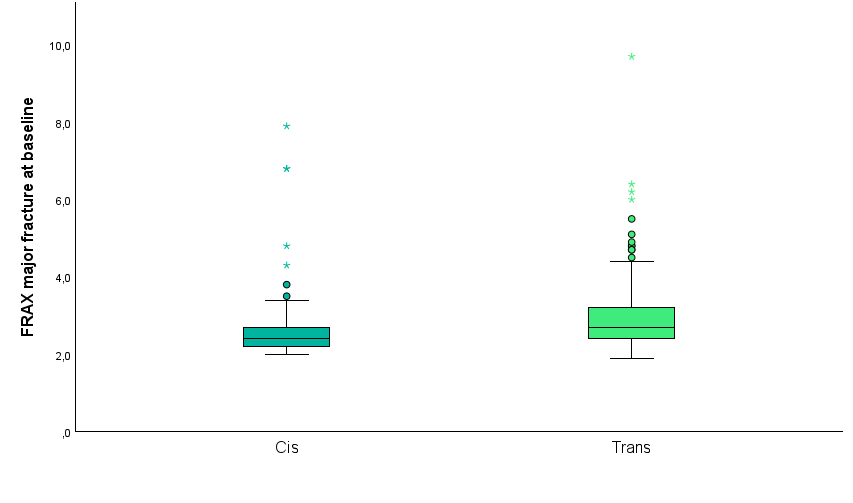


*p=0.01*


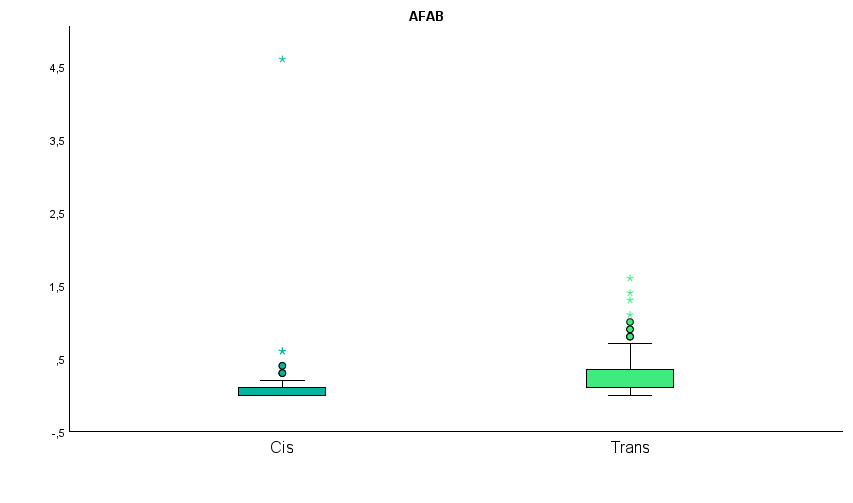

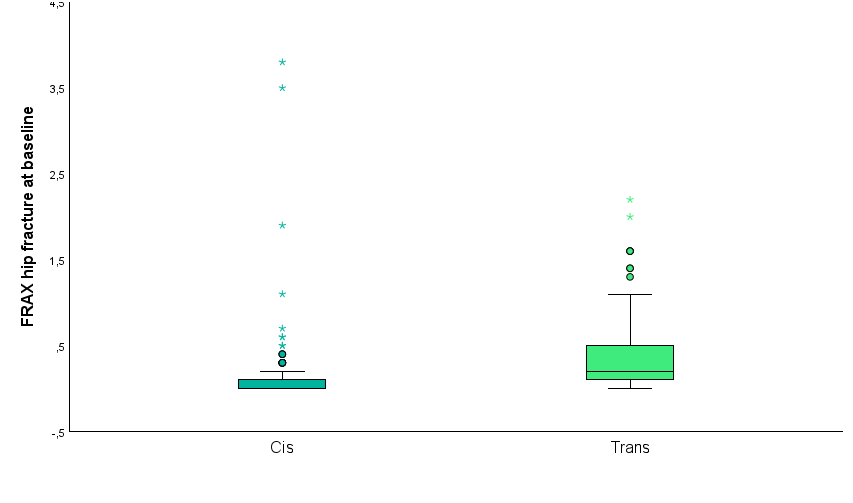

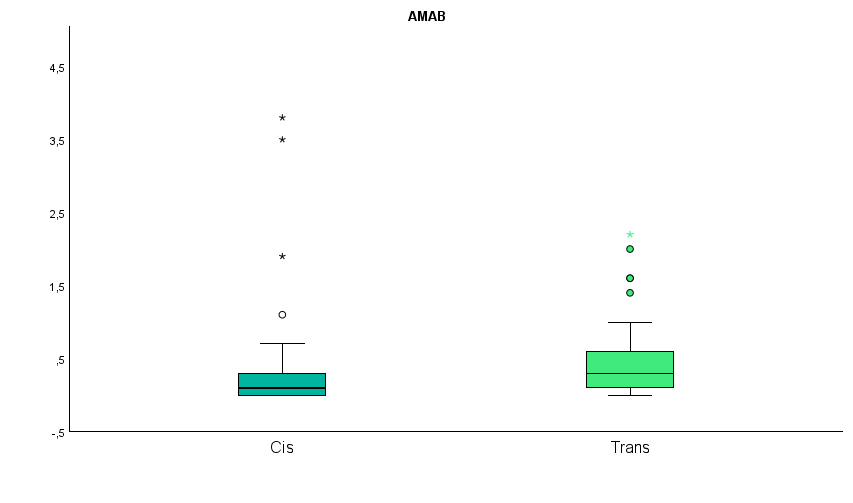


*p=0.01*
